# Outward Luxation of the Femur

**Published:** 1897-02

**Authors:** W. E. A. Wyman

**Affiliations:** Clemson Agricultural College, South Carolina


### OUTWARD LUXATION OF THE FEMUR.

BY W. E. A. WYMAN, V.S.,
CLEMSON AGRICULTURAL COLLEGE, SOUTH CAROLINA.

A FOUR-YEAR-OLD mule was brought to the free clinic with this history: Five weeks ago the animal, while playing in the mule-lot, stepped upon a corn-cob, fell, and was unable to rise. With some assistance he was gotten up, but the right hindleg was shorter than the left one. In a day or so decided swelling about the hip set in, which on applying a liniment(!) disappeared in about three weeks. The owner brought the animal to the clinic only to get a liniment to take the soreness out of the leg. Examination revealed the following: Inspection showed the leg abnormally adducted, the toe barely touching the ground, but supporting some weight; excessive atrophy of all the gluteal muscles; a prominence (palpation proving it to be the trochanter major of the femur) was seen postero-superior to the cotyloid cavity. Flexion of the stifle- and hock-joints limited, while abduction of the limb was quite impossible, the latter well marked during the futile attempts of the animal to kick the writer. Rectal examination was negative. The writer's diagnosis was rupture of the ligamentum teres and outward luxation of the femur (luxatio supra cotyloidea). Since the owner did not wish to kill the animal he was advised by one of the students to rub some liniment on the stable-door.

